# Supplementary material for: Dynamic filopodia are required for chemokine-dependent intracellular polarization during guided cell migration in vivo
Source: eLife. 2015 Apr 15;4:e05279. doi: 10.7554/eLife.05279 (PMC4397908; doi:10.7554/eLife.05279)
Supplement: Supplementary file 1. — DOI: http://dx.doi.org/10.7554/eLife.05279.039 [file elife05279s001.docx]

**Supplementary file 1A. Experimental Design**

| **Type of experiment** | **Fish lines used** | **Stage of injection** | **Material injected** | | | | | |
| --- | --- | --- | --- | --- | --- | --- | --- | --- |
| 10x- time-lapse movies (tracks) | *kop-egfp-f-nanos3’UTR* (EGFP labelling membranes of PGCs) | 1-cell | *mcherry_h2b_globin3'UTR* mRNA (mCherry labelling nuclei of all cells) + | | | | *pcd14_nos3'UTR* or  *dn irsp53_nos3'UTR* or  *afap1l1a_nos3'UTR* | |
| 10x- time-lapse movies (tracks) | homozygous Medusa *kop-egfp-f-nanos3’UTR*  (EGFP labelling membranes of PGCs) | 1-cell | *mcherry_h2b_globin3'UTR* mRNA (mCherry labelling nuclei of all cells) + | | | | *pcd14_nos3'UTR* or  *dn irsp53_nos3'UTR* | |
| 10x- time-lapse movies (tracks) | homozygous Medusa *kop-egfp-f-nanos3’UTR*  (EGFP labelling membranes of PGCs) | 1-cell | *mcherry_h2b_globin3'UTR* mRNA (mCherry labelling nuclei of all cells), *cxcr7bMO* + | | | | *pa_gfp_globin3'UTR or cxcl12a_globin3'UTR* | |
| 63x time-lapse movies  (filopodia dynamics and blebcount) | *kop-egfp-f-nanos3’UTR*  (EGFP labelling membranes of PGCs) | 1-cell | 1. uninjected | | | | | |
|  |  |  | *mcherry_h2b_globin3'UTR* mRNA (mCherry labelling nuclei of all cells) + | | | | *2. COMO* *or* *cxcr4b* MO or *ca15b* MO  3. *pcd14_nos3'UTR* or  *dn irsp53_nos3'UTR* or  *afap1l1a_nos3'UTR* | |
| 63x time-lapse movies (filopodia dynamics) | homozygous Medusa *kop-egfp-f-nanos3’UTR*  (EGFP labelling membranes of PGCs) | 1-cell | 1. uninjected | | | | | |
|  |  |  | *mcherry_h2b_globin3'UTR* mRNA (mCherry labelling nuclei of all cells), *cxcr7bMO* + | | | | 2. *pa_gfp_globin3'UTR* or *cxcl12a_globin3'UTR* | |
|  |  |  | *mcherry_h2b_globin3'UTR* mRNA (mCherry labelling nuclei of all cells) + | | | | 3. *pcd14_nos3'UTR* or  *dn irsp53_nos3'UTR* or *afap1l1a_nos3'UTR* | |
| 63x time-lapse movies  (F-actin content) | *kop-egfp-lifeact-nanos3’UTR; kop-mcherry-f-nanos3'UTR*  (EGFP labelling of F-actin, mCherry labelling membranes of PGCs) |  | uninjected | | | | | |
|  | homozygous Medusa *kop-egfp-f-nanos3’UTR*  (EGFP labelling membranes of PGCs) | 16-cell | lifeact_ruby_globin | | | | | |
| 63x time lapse movies  (Cxcl12a on filopodia) | *kop-mcherry-f-nanos3’UTR*  (mCherry labelling membranes of PGCs) | 1-cell and  16-cell | *cxcr7b* MO (1-cell) + | *cxcl12a_venus_globin3'UTR*  (16-cell) | | | | |
| 63x snapshots  (filopodia number) | *kop-egfp-f-nanos3’UTR*  (EGFP labelling membranes of PGCs) | 1-cell | *roc c'_nos3'UTR* (dn Rock) *+ mcherry_h2b_globin3'UTR* mRNA (mCherry labelling nuclei of all cells) + | | | | | *pcd14_nos3'UTR* or *rac1V12_nos3'UTR*  (ca Rac1) |
| 63x snapshots  (protein localization) | *kop-egfp-f-nanos3’UTR*  (EGFP labelling membranes of PGCs) | 1-cell | *mcherry_afap1l1a_nos3'UTR or irsp53_mcherry_nos3'UTR or*  *dn irsp53_mcherry_nos3'UTR* | | | | | |
| 63x snapshots (protein distribution and turnover) | AB (wild type) | 1-cell | 1. *cxcr4b_egfp_nos3'UTR + mcherry_f_nos3'UTR*  2. *cxcr4b_tft_nos3'UTR* | | | | | |
| Transplantation  (Cxcr4b distribution and turnover) | AB  **(donors)** | 1-cell | *cxcl12a-globin 3'UTR + cxcr7b* MO + *mcherry_h2b_globin3'UTR* mRNA (mCherry labelling nuclei of all cells) | | | | | |
|  | AB  **(hosts)** | 1-cell | *cxcl12a* MO *+ cxcr4b* MO + | | | 1. *cxcr4b_egfp_nos3'UTR + mcherry_f_nos3'UTR*  2. *cxcr4b_tft_nos3'UTR* | | |
| 63x snapshots (protein localization) | homozygous Medusa *kop-egfp-f-nanos3’UTR*  (EGFP labelling membranes of PGCs) | 1-cell | *cxcr4b_tft_nos3'UTR +* | | *pa_gfp_globin3'UTR* or *Cxcl12a_globin3'UTR* | | | |
| Counting % of ectopic PGCs | *kop-egfp-f-nanos3’UTR* (EGFP labelling membranes of PGCs) | 1-cell | *mcherry_f_nos3'UTR* (labelling nuclei of PGCs) + | | *1. pcd14_nos3'UTR* or  *dn irsp53_nos3'UTR afap1l1a_nos3'UTR*  *2. pa_gfp_globin3'UTR or cxcl12a_globin3'UTR* | | | |
| Rescue (ectopic PGCs) | homozygous ody | 1-cell | *egfp_f_nos3'UTR +* | | *pcd14_nos3'UTR* or  *cxcr4b_tft_nos3'UTR* | | | |
| pH-FRET | *kop-mCherry-f-nanos3’UTR* (mCherry labelling membranes of PGCs) | 1-cell | *pHlameleon5_nos3'UTR* (pH-sensor) + | | *pcd14_nos3'UTR* or  *dn irsp53_nos3'UTR afap1l1a_nos3'UTR* | | | |
| Rac1-FRET | *kop-mCherry-f-nanos3’UTR* (mCherry labelling membranes of PGCs) | 1-cell | *rac1FRET Ypet_noCT_ nos3’UTR* (Rac1 activity sensor) *+* | | *pcd14_nos3'UTR* or  *dn irsp53_nos3'UTR afap1l1a_nos3'UTR* | | | |
| Transplantation  (PGC response) | homozygous Medusa *kop-egfp-f-nanos3’UTR*  (EGFP labelling membranes of PGCs) **(donors)** | 1-cell | *mCherry-F-globin3'UTR*, *cxcr7bMO* + | | *cxcl12a-globin 3'UTR* or *pa_gfp_globin3'UTR* | | | |
|  | homozygous Medusa *kop-egfp-f-nanos3’UTR*  (EGFP labelling membranes of PGCs) **(hosts)** | 1-cell | uninjected | | | | | |
| Laser ablation | homozygous Medusa *kop-egfp-f-nanos3’UTR* (EGFP labelling membranes of PGCs) | 1-cell | *mcherry_f_globin3'UTR* | | | | | |

**Supplementary file 1B. Constructs Cloned for this Work**

| **Construct (internal number)** | **F-primer, 5’-3’** | **R-primer, 5’-3’** | **Amount injected, pg** |
| --- | --- | --- | --- |
| *irsp53_mcherry_nos3'UTR* (B922) | AAAAGATCTACCATGTCTCGCACCGACGAGGTC | AAAACTAGTCTGTGCAAAGCCTGCCATGCT | 100 |
| *DN_irsp53_nos3'UTR* (B519) | CGGATCCACCATGTCTCGCACCGACGAG | CGGATCCCACTGTGCAAAGCCTGCCAT | 600 |
| *DN_irsp53_mcherry_nos3'UTR* (C924) | GCCAGGCTGAGCTGGAGGAGCTGCGGGAGGAGAGCCAGGGCAGC | GCTGCCCTGGCTCTCCTCCCGCAGCTCCTCCAGCTCAGCCTGGC | 100 |
| *afap1l1a_nos3'UTR* (C252) | GGAAGATCTACCATGGAAATAAACAGCAAACC | CCGCTCGAGCTAAGTCCCCTTTTTAGAT | 600 |
| *mcherry_afap1l1a_nos3'UTR* (C879) | GGAAGATCTACCATGGAAATAAACAGCAAACC | CCGCTCGAGCTAAGTCCCCTTTTTAGAT | 200 |
| *cxcr4b_tft_nos3'UTR* (D013) | AGTGGGGATCCACCGGTCGCCACCATGGTGAGCAAGGGCGAGGA | TCAATGTCCGCTCTCGAGGCCGCTTTACTTATAAAGCTCGTCCATTCCGTG | 60, 100 |
| Whole mount *in situ* probe *irsp53* (D094) | CGAGCCTCATGGATGACCGATC | CCCTCTACTTACACCAGTGAAGATACGGATAC |  |
| Whole mount *in situ* probe *afap1l1a* (D093) | GGACGAGCACAGACGAAGCGTC | CACTCGCATATCATTCTGTAACATGGATC |  |

**Supplementary file 1C. Additional Constructs Used for this Work**

| **Construct (internal number)** | **Amount injected, pg** |
| --- | --- |
| *pcd14_nos3'UTR* (554) | Respective to the experimental RNA |
| *pa_gfp_globin3'UTR* (A918) | Respective to the experimental RNA |
| *mcherry_f_globin3'UTR* (A709) | 60 |
| *mcherry_f_nos3'UTR* (A906) | 100 |
| *mcherry_h2b_globin3'UTR* (B325) | 60 |
| *egfp_f_nos3'UTR* (493) | 120 |
| *cxcl12a_globin3’UTR* (642) | 2, 400 |
| *pHlameleon5_nos3'UTR* (B861) | 260 |
| *rac1FRET Ypet_noCT_ nos3’UTR* (A422) | 300 |
| *roc c'_nos3'UTR* (432) | 300 |
| *rac1V12_nos3'UTR* (481) | 200 |
| *cxcr4b_egfp_nos3'UTR* (760) | 200 |
| *cxcl12a_venus_gobin3'UTR* (B606) | 400 |
| *lifeact_ruby_globin3'UTR* (B852) | 140 |

**Supplementary file 1D. Morpholino Antisense Oligonucleotides Used for this Work**

| **Morpholino** | **Sequence 5’-3’** | **Concentration, mM** |
| --- | --- | --- |
| *ca15b* MO-2 | CCCTTTCAGTTTTTAACGATCACAC | 1 |
| *cxcr7b* MO | ATCATTCACGTTCACACTCATCTTG | 0.6/ 0.3 |
| *cxc4b* MO | TGCTCAAAAAGGTGCAATAAGTCCG | 0.3 |
| *cxcl12* MO | TTGAGATCCATGTTTGCAGTGTGAA | 0.2 |
| COMO | CCTCTTACCTCAGTTACAATTTATA | Respective to the experimental MO |

**Supplementary file 1E. FRET Analysis Protocol ImageJ**

NB: using images taken with 3 channels: CFP+YFP for FRET and red for mCherry membrane as a reference.

Prerequisites:

- PGC is the middle of the "run" phase
- (For pH-FRET) the front and the rear of the PGC are simultaneously in focus for at least 20 frames
- CFP signal is visible above the background

Procedure:

- open .lsm stack.
- convert to 32 bits: Image > Type > 32 bits.
- split channels: Image > Color > Split channels.
- (optional) correct for bleaching: Plugins > Macros > Correct bleach.
- create mask by using red channel:
  - - duplicate red channel: Image > Duplicate.
    - smooth image: Process > Filters > Gaussian Blur 2 pixels.
    - threshold: Image > Adjust > Threshold with option "Dark background". Reply "ok" to question "set background pixels to NaN".
    - convert to binary mask: Process > Binary > Convert to mask (with option "black background").
    - close any hole within the cell: Process > Binary > Fill holes.
    - smooth the edges: Process > Filters > Gaussian Blur 5 pixels. Then again Process > Binary > Convert to mask.
    - put background pixels to NaN:
    - convert to 32 bits: Image > Type > 32 bits
    - threshold: Image > Adjust > Threshold with option "Dark background". Reply "ok" to question "set background pixels to NaN".
- create CFP and YFP channels with NaN background: Process > Image Calculator, then multiply each channel by the mask.
- create the normalized FRET signal by dividing processed channel YFP by processed channel CFP: Process > Image Calculator

**For Rac1-FRET:**

- measure the average intensity of the whole cell

**For pH-FRET:**

- For front and back comparison: select region (Edit > Selection > Specify > oval, 35 pixel) and measure the intensity in the selected region in the front and in the back of the cell for 20 frames.
- divide the average value for the cell front intensities by the corresponding average value for the rear of the cell.

**Supplementary file 1F. Membrane Signal Analysis Protocol ImageJ**

NB: using images taken with 2 channels: GFP and mCherry.

For Cxcr4b distribution analysis Cxcr4b-EGFP is channel 1 and mCherry-F' is channel 2.

For Cxcr4b tft analysis mCherry is channel 1 and sfGFP is channel 2.

Prerequisites:

- PGC are polarized and in the "run" phase
- the front and the rear of the PGC are simultaneously in focus.

Procedure:

- open images of channel 1 and 2.
- subtract background: Rolling ball 50
- convert to 32 bits: Image > Type > 32 bits.
- Smooth image: Process > filters > Gaussian Blur 2 pixels
- create mask by using channel 2
  - - duplicate channel: Image > Duplicate
    - threshold: Image > Adjust > Threshold with option "Dark background". Reply "ok" to question "set background pixels to NaN".
    - convert to binary mask: Process > Binary > Convert to mask (with option "black background").
    - convert to 32 bits: Image > Type > 32 bits.
    - threshold: Image > Adjust > Threshold with option "Dark background". Reply "ok" to question "set background pixels to NaN".
- create GFP and mCherry channels with NaN background: Process > Image Calculator, then multiply each channel by the mask.
- create the normalized data by dividing processed channel 1 by processed channel 2 (which was used for the mask): Process > Image Calculator
- select region (select segmented line with thickness 5) and measure the mean intensity in the selected region in the front and in the back of the cell of the first frame.
